# Pulsed Laser Deposition of Nanostructured MoS_3_/np-Mo//WO_3−*y*_ Hybrid Catalyst for Enhanced (Photo) Electrochemical Hydrogen Evolution

**DOI:** 10.3390/nano9101395

**Published:** 2019-09-30

**Authors:** Vyacheslav Fominski, Alexey Gnedovets, Dmitry Fominski, Roman Romanov, Petr Kartsev, Oxana Rubinkovskaya, Sergey Novikov

**Affiliations:** 1 National Research Nuclear University MEPhI (Moscow Engineering Physics Institute), Moscow 115409, Russia; dmitryfominski@gmail.com (D.F.); limpo2003@mail.ru (R.R.); pfkartsev@mephi.ru (P.K.); oxygenofunt@gmail.com (O.R.); 2 Baikov Institute of Metallurgy and Materials Science, Russian Academy of Sciences, Moscow 119334, Russia; a.gnedovets@hotmail.com; 3 Moscow Institute of Physics and Technology, Moscow 141700, Russia; Novikov.S@mipt.ru

**Keywords:** pulsed laser deposition, nanocatalysts, background gas, tungsten oxides, transition metal chalcogenides, hydrogen evolution reaction

## Abstract

Pulsed laser ablation of MoS_2_ and WO_3_ targets at appropriate pressures of background gas (Ar, air) were used for the preparation of new hybrid nanostructured catalytic films for hydrogen production in an acid solution. The films consisted of a nanostructured WO_3−*y*_ underlayer that was covered with composite MoS_3_/np-Mo nanocatalyst. The use of dry air with pressures of 40 and 80 Pa allowed the formation of porous WO_3−*y*_ films with cauliflower- and web-like morphology, respectively. The ablation of the MoS_2_ target in Ar gas at a pressure of 16 Pa resulted in the formation of amorphous MoS_3_ films and spherical Mo nanoparticles. The hybrid MoS_3_/np-Mo//WO_3−*y*_ films deposited on transparent conducting substrates possessed the enhanced (photo)electrocatalytic performance in comparison with that of any pristine one (MoS_3_/np-Mo or WO_3−*y*_ films) with the same loading. Modeling by the kinetic Monte Carlo method indicated that the change in morphology of the deposited WO_3−*y*_ films could be caused by the transition of ballistic deposition to diffusion limited aggregation of structural units (atoms/clusters) under background gas pressure growth. The factors and mechanisms contributing to the enhancement of the electrocatalytic activity of hybrid nanostructured films and facilitating the effective photo-activation of hydrogen evolution in these films are considered.

## 1. Introduction

Water splitting under the electric current, visible light, or the simultaneous effects of electric current and light, is one of the most attractive ways to produce hydrogen. The effective and low-cost production of this gas energy source without spending natural hydrocarbon raw materials largely determines the successful development and implementation of alternative (green/hydrogen) energetics. A significant reduction in energy consumption during the hydrogen evolution by photo-activated electrolysis is dependent on the success of the development and creation of new efficient non-precious electrocatalysts and photo-electrocatalysts, the manufacture of which does not use expensive materials (platinum group metals) [1,2,3,4,5,6]. Recently, a motivated interest has been formed in nanostructured and hybrid materials containing chalcogenides, phosphides, nitrides, carbides, or oxides of transitional metals [7,8,9,10,11,12]. To obtain effective electrocatalysts based on these materials for activating the hydrogen evolution reaction (HER), it is necessary to solve the problems of adjusting their structural and chemical states at the nanoscale, or even at a level of atomic arrangement [13,14,15]. It is also important to reasonably choose the support material for the nanocatalysts that cause a synergistic influence on the electrocatalytic HER or provide a significant increase in the efficiency of nanocatalysts under the visible light illumination, e.g., due to enhanced separation of photo-induced electron-hole pairs in heterostructures [16,17,18].

Molybdenum sulfide (MoS*_x_*) is a prominent representative of promising (photo)electrocatalysts for the hydrogen evolution in acid and alkaline solutions [19,20]. For use in acidic solutions, the MoS*_x_* catalysts with an amorphous structure and higher sulfur content (*x* > 2) is especially relevant [21,22,23,24,25]. To obtain the MoS*_x_*-based catalyst, chemical (hydrothermal or electrochemical) processes are mainly used. Tungsten oxide (WO_3−*y*_) can also exhibit quite perfect (photo)electrocatalytic properties for HER activation, however, certain requirements for the structure and phase composition should be realized [9,15,26]. For the preparation of a high-quality metal oxide catalyst, a method is needed that makes it possible to flexibly modify the structural, morphological and chemical performances of the catalyst. Obviously, the requirement for the method of producing catalysts of this type increases when hybrid catalysts should be formed. The formation of hybrid nanostructures containing chalcogenides and oxides of transition metal should be considered as a perspective direction for obtaining new (photo)cathodes for enhanced (photo-assistant) electrochemical HER [9,16,27,28,29].

Pulsed laser deposition (PLD) is a universal method for producing various nanomaterials. However, when creating efficient/advanced nanocatalysts based on molybdenum sulfide and tungsten oxide, several problems arise. Upon laser ablation of the MoS_2_ target, the erosion plume contains not only electrons and atoms (including ionized ones), but also particles of submicron and nanometer sizes [30,31,32]. The particles of nanometer size have a rounded shape and consist of crystalline molybdenum. The effect of these particle deposition on electrocatalytic properties of molybdenum sulfide films is not well understood. 

During laser ablation of the targets made of transitional metal dichalcogenides under vacuum conditions, the bombardment of the deposited layers by high-speed atoms cause a preferential sputtering of chalcogen atoms (S, Se) that results in the formation of a substoichiometric compound (*x* = Chalcogen/Metal < 2) [33,34,35]. The use of background gas during PLD makes it possible to increase the chalcogen atoms concentration in the films [33,36]. However, the (photo)electrochemical HER characteristics of S-enriched MoS*_x_*_>2_ films containing Mo nanoparticles in hybrid nanostructures with tungsten oxide support have not yet been studied. It should be noted that application of the reactive PLD allows the variation of the S concentration in MoS*_x_* films in a wide range [37]. However, the reactive PLD requires the use of hazardous and harmful gas (H_2_S). Therefore, the problem of obtaining highly S-enriched MoS*_x_*_≈ 3_ films by the simplest method of pulsed laser ablation of the MoS_2_ target remains relevant.

The use of a background gas during the PLD of WO_3−*y*_ films facilitates the preparation of the films possessing various structures and morphology [38,39,40,41,42]. Preliminary studies of the different WO_3−*y*_ films, which were obtained by the reactive PLD from Mo target, showed that they did not exhibit high electrocatalytic activity in HER [43]. However, these nanostructured films have been successfully used as supports for Pt nanoparticles. The HER activity of a hybrid Pt/WO_3−*y*_ nanocatalyst was better than that of a metallic Pt film. The effect of WO_3−*y*_ films on the electrocatalytic activity of MoS*_x_*_≈ 3_ films enriched in sulfur has not been carefully studied. There are no data on the use of a MoS*_x_*_≈ 3_/WO_3−*y*_ hybrid for photoelectrochemical HER. The use of WO_3−*y*_ films for photocatalytic hydrogen evolution is controversial, since the lower edge of the conduction band of this semiconductor lies below the potential of the hydrogen reduction [9]. Therefore, MoS*_x_*/WO_3−*y*_ hybrid photocatalyst can operate only as a two-step excitation system (the Z-scheme), in which MoS*_x_* layer catalytically activates HER. 

The main aim of the work was to create a hybrid (photo)electrocatalytic structure (MoS_3_/np-Mo//WO_3−*y*_) based on a nanostructured WO_3−*y*_ support-film and a thin MoS_3_ catalytic film containing Mo nanoparticles (np-Mo). The WO_3_ and MoS_2_ targets were used for the preparation of these films by PLD. To obtain the porous WO_3−*y*_ films with very different morphologies and the S-enriched composite MoS_3_/np-Mo films, special PLD regimes in background gases of relatively high pressures were applied. 

For the hybrid nanostructured MoS_3_/np-Mo//WO_3−*y*_ film obtained under optimal conditions, the enhanced (photo)electrocatalytic performance in HER reaction was achieved. The experimental/structural studies and modelling by the kinetic Monte Carlo method indicated that a background gas (dry air) affects the transport mechanism of laser-initiated plume and facilitates the growth of a cauliflower-like morphology of WO_3−*y*_ films at a pressure of 40 Pa and a web-like structure of these films at a higher pressure (80 Pa). The amorphous WO_3−*y*_ films possessing cauliflower-like morphology most effectively influenced the (photo)electrocatalytic performances of the hybrid films. Thermodynamic analysis using density functional theory (DFT) has shown that enhancement of electrocatalytic performance of hybrid films could be caused by synergistic effect of MoS_3_/Mo and MoS_3_/WO_3_ interfaces. The photoelectrochemical HER of the hybrid structure was probably initiated due to realization of Z-scheme of electron and hole separation between WO_3−*y*_ and MoS_3_ semiconductors under visible light illumination.

## 2. Materials and Methods 

### 2.1. Experimental Methods and Materials

The WO_3_ and MoS_2_ targets were ablated by nanosecond pulses from a Solar LQ529 laser (Solar LS, Minsk, Belarus) with radiation wavelength of 266 (ultraviolet radiation, UV) and 1064 nm (infrared radiation, IR), respectively. Preliminary studies have shown that the choice of UV radiation is of great importance for WO_3_ target ablation. When using IR-radiation for this target ablation, a large number of particles of submicron/micron sizes can be formed and deposited on the films and degrade their properties. Our comparative studies of the ablation of the MoS_2_ target by laser pulses with UV and IR radiation indicated that the wavelength did not significantly affect the particle formation. Therefore, the use of IR radiation is energetically beneficial. Besides, due to the higher energy of the laser generating IR pulses, the possibilities of variation of ablation regimes for the MoS_2_ target increase markedly.

The energy of laser pulses was approximately 20 mJ, the laser fluence was ~5 J/cm^2^ and pulse repetition rate was 20 Hz. The selected parameters provided a weak erosion of the target surface when the target was exposed to a single laser pulse. During the PLD processes, the targets were moved in two directions that enabled the maintenance of a relatively smooth surface of the targets in the ablation zone under the repeatable laser ablation. The targets were made by pressing WO_3_ and MoS_2_ powders with particle sizes of several micrometres.

For preparation of the WO_3−*y*_ films, the deposition of laser-ablated plume from the WO_3_ target was performed in dry air at pressures of 40 and 80 Pa. The use of air as a reactive background atmosphere makes it possible to increase the saturation efficiency of the deposited WO_3−*y*_ film with oxygen atoms. There is also an important problem in identifying the feasibility of forming WO_3−*y*_ film with desired properties from W and WO_3_ targets in reactive and inert background atmospheres. In the case of reactive PLD in air, the issue of the influence of air humidity on the properties of films is relevant. Obviously, the PLD in air of the laboratory humidity is most attractive in terms of simplicity and low cost of technological processes. This work used dry air for the WO_3−*y*_ film formation from the WO_3_ target. Previously, the characteristics of WO_3−*y*_ films formed by PLD from W target in rarefied wet air were considered by the authors in [41].

The deposition of MoS_3_/np-Mo film was conducted in Ar background gas at a pressure of 16 Pa. Our experience in the field of PLD of MoS*_x_* films in reactive and inert background atmosphere asserted that Ar molecules efficiently scatter the flux of Mo and S atoms propagating with a laser plume [32,33]. The use of this gas does not cause noticeable contamination of the films. For Ar pressure of 16 Pa, important changes in the structure and composition of the MoS*_x_* films deposited from the MoS_2_ target could appear. Obviously, the use of reactive H_2_S gas is a more effective process to control the chemical composition of the MoS*_x_* films obtained by reactive PLD [37]. However, this gas is harmful and dangerous. 

To implement sufficiently clean deposition conditions, the vacuum chamber for PLD was previously pumped out with a turbomolecular pump to a pressure of 10^−4^ Pa. Then, the required background gas was injected into the chamber to a predetermined pressure. Deposition was performed in a small chamber with a volume of ~500 cm^3^ that made it possible to increase the partial pressure of the sulphur vapor around the substrate during the ablation of the MoS_2_ target. The PLD processes duration for WO_3−*y*_ and MoS_3_/np-Mo films were 20 and 3 min, respectively. In a special case, the deposition time of MoS_3_/np-Mo film was changed. After the deposition, some WO_3−*y*_ films were subjected to thermal posttreatment (annealing) at 500 °C for 1 h in air.

The features of the laser plumes that expanded from WO_3_ and MoS_2_ targets under different background pressures were studied by collecting plasma plume images through the viewport (orthogonal to the axis of the plume transport). A digital camera with an exposure time of 0.5 s was used. This time corresponded to an average value of more than 20 pulses. The different plumes were assumed to be equivalent. Therefore, the times, shapes, and sizes of the integrated plumes (i.e., the visible parts) were quite adequately recorded. Further, the measurements of the time-of-flight ion pulses of laser plasma by the ion probe placed near the substrate were performed. A negative bias of 40 V was applied to the probe for reflection of the laser-plasma electrons.

Furthermore, 0.5 mm-thick glass plates covered with fluorine doped tin oxide (FTO) coating were used as substrates for catalytic films. The dimension of the PLD covered area of the substrate used for electrochemical investigation was 0.5 × 1 cm^2^. The surface morphologies of the WO_3−*y*_ films prepared on the FTO substrates were studied using scanning electron microscopy (SEM, Tescan LYRA 3, Czech Republic). The structures of these films were examined by micro-Raman spectroscopy (MRS) using a 632.8-nm (He-Ne) laser. The cross section of the laser beam was < 1 μm.

The catalyst loadings as well as O/W and S/Mo atom content ratios were measured by Rutherford backscattering spectroscopy (RBS) (He ion energy, 1.3–2.5 MeV; scattering angle, 160°). For this study, the WO_3−*y*_ and MoS_3_/np-Mo films were formed by PLD on polished SiC and Si substrates under the conditions which were used for preparation of the catalytic films on the FTO substrates. Mathematical modelling of the RBS spectra was performed using the SIMNRA code [44]. 

To study the structures and composition of MoS_3_/np-Mo catalyst, the films were formed by PLD on Si and NaCl crystals. The films deposited on the Si substrate were investigated by the grazing incidence X-ray diffraction (XRD, Cu Kα radiation, Ultima IV, Rigaku, Tokyo, Japan) and X-ray photoelectron spectroscopy (XPS, K-Alpha apparatus, Thermo Scientific, Madison, WI 53711, USA) with Al Kα radiation (1486.6 eV). NaCl crystals with a deposited film were dipped in distilled water. After separation of the films from the NaCl substrate, the films were placed on fine-grained metal grids and transferred to an electron microscope (Carl Zeiss Libra 120 equipped with EDXS X-MAX 80T (Oxford Instruments, UK)) for transmission electron microscopy (TEM), selected-area electron diffraction (SEAD) and energy dispersive X-ray spectroscopy studies (EDXS). 

The studies of the electrocatalytic HER performance of MoS_3_/np-Mo//FTO, WO_3−*y*_//FTO and MoS_3_/np-Mo//WO_3−*y*_//FTO samples were conducted in 0.5 M H_2_SO_4_ aqueous solution (after purging with H_2_) using an Elins Instruments electrochemical analyser (Model P-5X, Chernogolovka, Russia). A saturated silver chloride electrode (Ag/AgCl) was used as the reference electrode, and the prepared sample was the working electrode. A glassy carbon plate was used as a counter electrode. All potentials reported in this work were measured versus the reversible hydrogen electrode (RHE), and they were calculated according to the following formula: *U*(RHE) = *U*(Ag/AgCl) + (0.205 + 0.059 pH) (pH ≈ 0). For FTO substrates with catalytic films, the polarisation curves were measured using linear sweep voltammetry (LSV) with a change of the applied potential from 0 to −500 mV and a scan rate of 2 mV/s. Electrochemical impedance spectroscopy was performed to estimate the equivalent series resistance (*R*_s_) of the samples. The *iR*_s_-correction of LSV curves was applied only in special cases.

To study the photoelectrocatalytical properties of the prepared samples, they were illuminated by radiation of Xe lamps with 150 W power in 0.5 M H_2_SO_4_ aqueous solution. After the UV filter, the wavelength range of the lamp was ~420–830 nm. The light intensity was maintained at 100 mW/cm^2^, which was equivalent to the irradiance of one solar constant (one sun). The lamp light was focused on the sample by a spherical lens and the area of illumination was ~1 cm^2^. A three-electrode configuration was used to measure the photo-activated current in an electric circuit with modified cathodes. The potential of the tested samples was maintained at zero level (relative to RHE).

### 2.2. Computational Methods

The film structure evolution was simulated by the kinetic Monte Carlo (KMC) method on the basis of modified Vold-Sutherland [45] and Witten-Sander [46] algorithms in ballistic deposition (BD) and diffusion-limited aggregation (DLA) modes for the relatively low and high buffer gas pressures, respectively. A detailed description of the simulation setup can be found elsewhere [47]. Briefly, the film growth was considered on a three-dimensional simple cubic lattice *L_X_* × *L_Y_* × *L_Z_* with periodic boundary conditions along the *X* and *Y* directions parallel to the substrate surface. In the BD regime, the incident deposited particles followed random straight-line trajectories, while in DLA mode, they performed off-lattice Brownian random walks with a given step length until they collided with the substrate or film surface. All KMC experiments have been conducted for *L_X_* = *L_Y_* = *L_Z_* = 512*a*, where *a* is the size of the elementary structural block (atom or cluster) of the film.

To reveal the influence of Mo nanoparticles on the HER activity of MoS_3_ electrocatalyst and the possibility of synergistic interaction of MoS_3_ thin films with a WO_3−*y*_ underlayer, a DFT simulation of several simplified atomic structures using the quantum ESPRESSO package [48] was performed. The details of the simulation and the parameters used for the calculations of the change of Gibbs free energy (*G_H_*) for hydrogen adsorption on the active sites of catalysts are given in Appendix A.

## 3. Results

### 3.1. Characterization of Pulsed Laser Plumes Formed during Ablation of WO_3_ and MoS_2_ Targets in Background Gases

**Figure 1:**
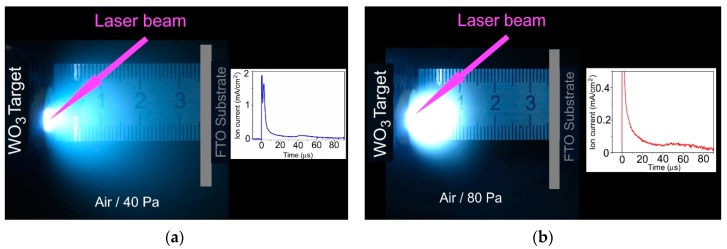
Time integrated optical images of laser plumes formed during pulsed laser ablation of the WO_3_ target at dry air pressures of (**a**) 40 Pa and (**b**) 80 Pa. The arrangement of fluorine doped tin oxide (FTO) substrates with respect to the axis of laser plume expansion is shown. The inserts show pulsed ion signals detected by ion probe at the place of substrate location.

Figure 1 shows the results of the study for pulsed laser plume initiated by pulsed laser ablation of the WO_3_ target under two pressures of dry air. In the case of 40 Pa pressure, a noticeable limitation of the plume volume was observed, but the front of its expansion touched the substrate surface. For a pressure of 80 Pa, the size of the plume glow area was markedly less than the distance from the WO_3_ target to the substrate. The measurements of ion signals for laser plumes indicated that an increase in air pressure caused both a decrease in the intensity of ion bombardment and a noticeable change of the ion energy (inserts in Figure 1). For a pressure of 40 Pa, along with a peak from the high-speed ions (the time of ion flight is ~10 μs), a peak with the time of ion flight of more than 40 μs appeared. For a pressure of 80 Pa, the ion signal was very weak, and it consisted of one broadened peak arising 45 μs after pulsed laser irradiation of the WO_3_ target.

**Figure 2:**
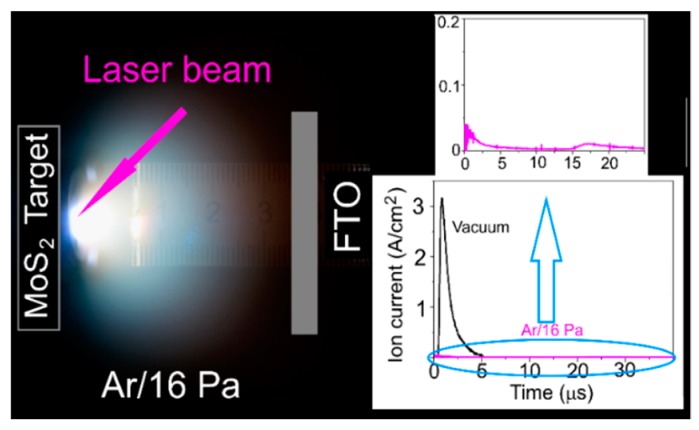
Time integrated optical image of laser plume formed during pulsed laser ablation of the MoS_2_ target in Ar at a pressure of 16 Pa. The inserts show pulsed ion signals detected by ion probe at the place of FTO substrate location. The pulse of high-speed ions measured under vacuum conditions of pulsed laser ablation of this target is shown for comparison.

The results of study of laser plume from the MoS_2_ target are shown in Figure 2. At a selected Ar pressure (16 Pa), the character of the expansion of the plume from this target coincided, to some extent, with that of the plume from the WO_3_ target at the highest pressure of dry air. The laser plume stopped in front of the substrate and the high-speed ions did not bombard the growing film. The ion detector recorded only the signal from low-speed ions losing their kinetic energy in collisions with Ar molecules. A broad peak with low intensity appeared after laser irradiation of the MoS_2_ target with a 15 μs delay. Obviously, this peak arises, first of all, due to the interaction of the heaviest atoms in the laser plume with background gas molecules that results in collective movement of atoms and molecules. The difference in the mass of Mo and W atoms, and consequently, the difference in their speeds of the movement in the laser plume, caused the difference in the delay times of ion signals in the cases of pulsed laser ablation of the WO_3_ and MoS_2_ targets.

### 3.2. Composition and Structure of WO_3−y_ and MoS_3_/np-Mo Films

**Figure 3:**
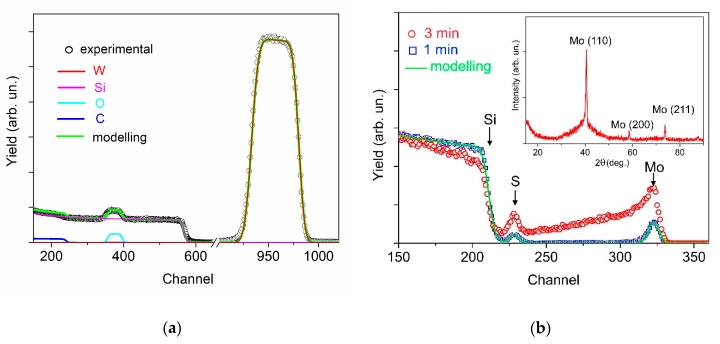
The results of Rutherford backscattering spectroscopy (RBS) studies: (**a**) Experimental and model spectra for WO_3−*y*_ film deposited on the SiC substrate at air pressure of 40 Pa; (**b**) experimental and model spectra for MoS_3_/np-Mo films deposited on the Si substrate at Ar pressure of 16 Pa for 1 and 3 min. The insert shows X-ray diffraction (XRD) spectrum for thicker MoS_3_/np-Mo film.

Figure 3 shows the RBS spectra for WO_3−*y*_ and MoS_3_/np-Mo films deposited on SiC and Si substrates, respectively. The conditions/regimes of the films deposition completely coincided with those used for preparing the hybrid MoS_3_/np-Mo//WO_3−*y*_ film on the FTO substrate. The experimental RBS spectrum for the WO_3−*y*_ film on SiC may be quite correctly processed by the SIMNRA program (Figure 3a).

In accordance with the SIMNRA deconvolution of the experimental RBS spectrum for WO_3−*y*_ film, the value of atomic ratio O/W was approximately 2.9 ± 0.1. The surface atomic density of the films was estimated to be ~10^18^ atom/cm^2^. This value corresponds to the thickness of ~140 nm for the film possessing tabulated density (7.16 g/cm^3^). SEM studies of the cross-section image of this film revealed that the thickness was ~300 nm (It is shown below). This indicated a high porosity of the WO_3−*y*_ film prepared by PLD at air pressure of 40 Pa. The RBS study of the WO_3−*y*_ film deposited at air pressure of 80 Pa revealed practically the same composition of the film. However, the deposition rate of this film was two times lower than that at a pressure of 40 Pa.

The RBS spectrum of the MoS_3_/np-Mo film shown in Figure 3b had specific features which excluded a possibility of its precision processing with SIMNRA. The signal from He ions scattered by Mo atoms had a long tail in the low-energy region (channels region is 225–325) and overlapped with a peak from He ions scattered by S atoms (channels region is 220–230). This feature of the spectrum indicates the presence of a large number of Mo nanoparticles deposited with a thin MoS_3_ film. The sizes of these Mo particles exceeded the thickness of MoS_3_ film. Indeed, on the XRD spectrum of this film, in addition to a diffuse halo belonging to amorphous phase, there were lines corresponding to the reflection from cubic crystal lattice of molybdenum (insert in Figure 3b).

To evaluate the MoS_3_ film thickness and measure its composition, additional experiments were conducted. A thinner film with a lower content of Mo particles was obtained by PLD when the deposition time was reduced by three times. The result of the RBS study of a thinner MoS*_x_* films is shown in Figure 3b. SIMNRA modelling of the experimental spectra allowed the estimation of the atomic ratio *x* at ~3.0 ± 0.1, and the deposition rate of this MoS_3_ film was approximately 1.5 μg/cm^2^/min. Therefore, the loading of the MoS_3_ catalyst for 3 min of PLD did not exceed 4.5 μg/cm^2^. At a density of ~5 g/cm^3^, ~9 nm thick MoS_3_ film corresponded to this loading.

The described above result of *x* measurement by RBS correlated reasonably well with that obtained by XPS for the deposited MoS_3_/np-Mo films. The Mo to S ratio of 1 to 3.2 was determined by XPS quantification. It was previously found that the data of S content on the surface of MoS*_x_* films obtained by XPS may be overestimated in comparison with those obtained by RBS measurements [33]. The S adsorption on the surface of the film in a vacuum chamber after MoS_3_/np-Mo film production by PLD is one of the possible reasons for this fact.

**Figure 4:**
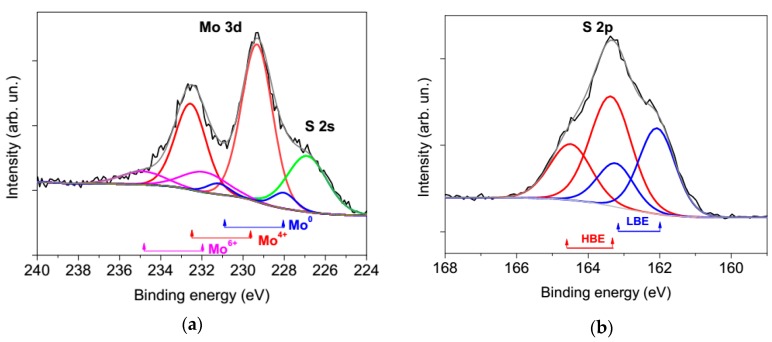
Experimental and modelling X-ray photoelectron spectroscopy (XPS) spectra of MoS_3_/np-Mo film: (**a**) Mo 3d and (**b**) S 2p spectra measured on the surface of the film. The main chemical states of Mo (molybdenum valences) and S atoms with a low (LBE) and high binding energy (HBE) are shown.

Figure 4 shows the XPS Mo 3d and S 2P spectra. These measured on the surface of the MoS_3_/np-Mo film deposited on the Si substrate. In the spectrum of Mo 3d, in addition to the doublet Mo 3d_5/2_–Mo 3d3_/2_, which corresponds to the chemical bonding of Mo with S (Mo^4+^, the binding energy E_B_ of Mo 3d_5/2_ is 229.7 eV), there were two doublets that were attributable to metallic Mo (Mo^0^, Mo 3d_5/2_ E_B_ ≈ 228 eV) and Mo oxide (Mo^6+^, Mo 3d_5/2_ E_B_ ≈ 232.8 eV). The spectrum of metallic Mo indicated the presence of Mo nanoparticles that were not covered with the MoS_3_ shell or/and the shell around some particles was very thin. The metal oxide formation on the film surface could proceed due to a slow transformation from Mo^4+^ to Mo^6+^ under atmospheric conditions [23]. This process could have partially occurred between the preparation and characterisation of the MoS*_x_* thin films.

The S 2p spectrum consisted of two doublets with S 2p_2/3_ binding energies of 162.1 (LBE, low binding energy) and 163.4 eV (HBE, high binding energy). For MoS_3_/np-Mo film deposited at Ar pressure of 16 Pa, the relative contribution of the HBE doublet in the S 2p spectrum significantly exceeded that for MoS*_x_* films prepared by PLD under vacuum conditions [33]. This indicated the formation of specific S ligands in the films deposited in a background atmosphere that can facilitate the enhanced HER activity. The same XPS spectrum was observed previously for amorphous MoS_3_ prepared by electro-polymerization procedures [21]. The HBE doublet might be attributed to the bridging S_2_^2−^ and/or apical S^2−^ ligands. The S ligands with LBE are assigned to the S_2_^2−^ terminal and/or unsaturated S^2−^ entities in the amorphous MoS*_x_* and the S^2−^ in the crystalline MoS_2_.

**Figure 5:**
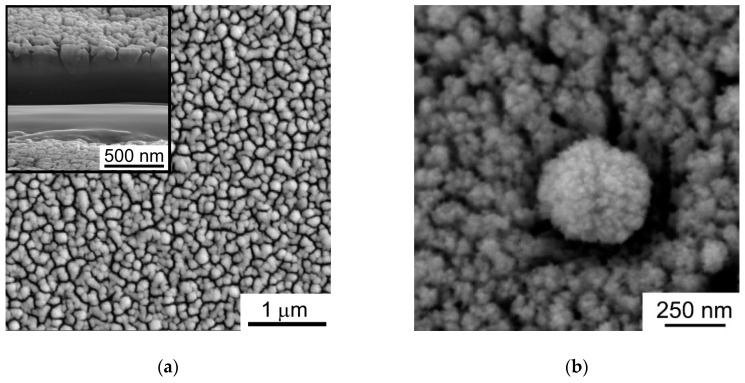
Scanning electron microscopy (SEM) images of the WO_3−*y*_ films deposited on FTO substrate at air pressure of 40 Pa: (**a**) the area without particles; (**b**) the area containing a nanoparticle which was covered with WO_3−*y*_ shell by pulsed laser deposition (PLD). Insert in (**a**) shows a cross section of local area formed by film thinning with focused ion beam.

**Figure 6:**
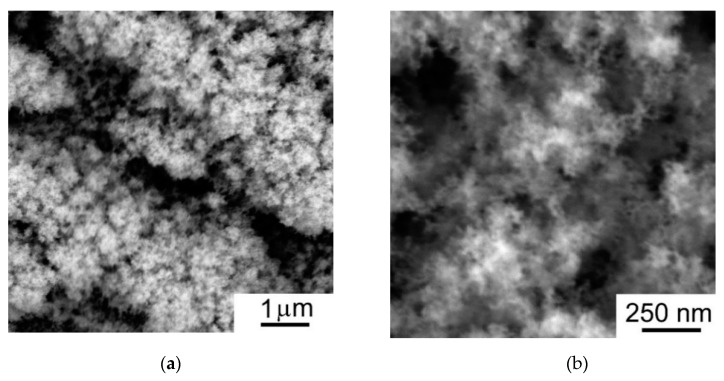
SEM images (with two magnifications) for the WO_3−*y*_ film prepared by PLD at dry air pressure of 80 Pa: (**a**) low and (**b**) higher magnifications.

The SEM studies of WO_3−*y*_ films indicated the marked difference in morphology of the films deposited at air pressures of 40 and 80 Pa (Figure 5 and Figure 6). In the case of 40 Pa pressure, a cauliflower-like morphology was formed. WO_3−*y*_ films grew from the surface of the substrate in the cauliflower columnar form with crowns expanding at the film surface. The crowns could cause a shadow effect which clearly manifested when the WO_3−*y*_ film was deposited on the nanoparticle shown in Figure 4b. The core-particle was probably deposited from the laser plume at the initial stage of the WO_3−*y*_ film growth. As a result of the shadow effect, a depleted region was formed in the environment of the particle. The atoms from the laser plume did not penetrate in this region. The morphology of the WO_3−*y*_ film prepared at air pressure of 80 Pa may be characterized as web-like [39]. This suggests that the increase of air pressure significantly alters the growth mechanism of WO_3−*y*_ films. 

**Figure 7:**
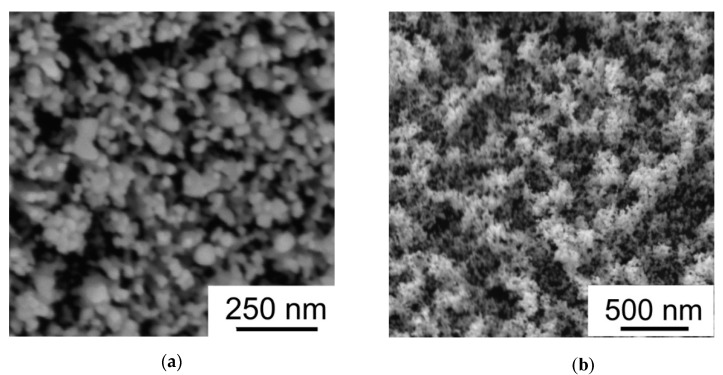
SEM images measured after thermal posttreatment of the films in air at 500 °C: (**a**) WO_3−*y*_ film deposited at air pressure of 40 Pa, (**b**) WO_3−*y*_ film deposited at air pressure of 80 Pa.

**Figure 8:**
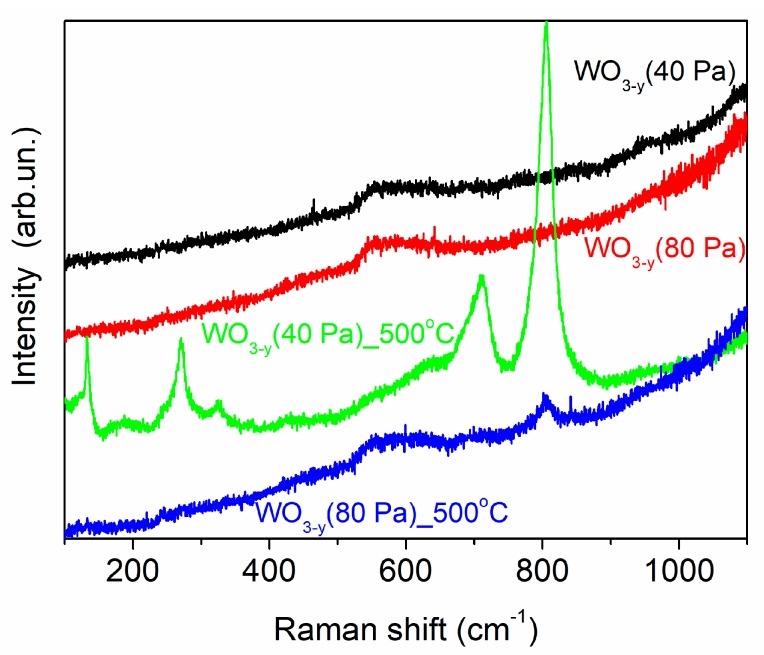
Micro-Raman spectra for the WO_3−*y*_ films prepared on FTO substrates at dry air pressures of 40 Pa and 80 Pa. The spectra were measured before and after thermal posttreatment in air at 500 °C.

The SEM studies of annealed WO_3−*y*_ films showed that morphologies of the films did not change markedly after thermal posttreatment (Figure 7). However, Raman studies revealed a tendency to local structural ordering (crystallization) in these films during thermal annealing. Figure 8 shows the Raman spectra that were measured before and after thermal annealing of the WO_3−*y*_ films obtained on FTO substrates at two pressures of dry air. The results of Raman studies indicated that the PLD of WO_3−*y*_ films at room temperature resulted in the formation of an amorphous structure independently on background gas pressure. The Raman spectra contained no lines that were characteristic of any crystalline WO_3_ structures. Only a broad peak located at ~560 cm^−1^ was detected before the WO_3−*y*_ films annealing. This peak appeared due to the penetration of laser radiation into a deep layer of the samples and resonance reflection of the laser radiation from the FTO substrate [49].

The thermal posttreatment at 500 °C in air of laboratory humidity (~50% relative humidity) caused a significant structural change for the amorphous WO_3−*y*_ film preliminary deposited at a pressure of 40 Pa. After posttreatment, the Raman spectra of this film contained the main lines characteristic of polycrystalline structure. The peak at 130 cm^−1^ belongs to the W–O–W bending modes, and peaks at 270 and 328 cm^−1^ are assigned to the O–W–O bending modes. The two peaks at ~700 and 806 cm^−1^ belong to the W–O stretching modes. These lines are characteristic for both the stoichiometric WO_3_ and the substoichiometric WO_3−*y*_, if a rather ordered WO_6_ octahedra packing are realized in the structure [41,50,51]. The peaks broadened and an almost imperceptible peak at 328 cm^−1^ indicated a satisfactory symmetry of atomic packing and a large quantity of oxygen vacancies in the WO_3−*y*_ film.

The Raman spectra in Figure 8 illustrated that thermal posttreatment of the WO_3−*y*_ film, which was preliminary deposited at higher air pressure, resulted in a weak structural modification. Only two broad and low-intensity peaks at ~704 and ~804 cm^−1^ arise on the Raman spectra after annealing. This suggests that after thermal posttreatment, the web-like WO_3−*y*_ films retained structural disorder of a relatively high degree. It may be caused by very small dimensions of structural units in the web-like material that prevent the growth of larger crystals. The thermal treatment conditions of these very porous films may have specific features arising from a very low thermal conductivity of such structure and a high thermal resistance of the contact area of the web-like WO_3−*y*_ film with the FTO substrate. 

**Figure 9:**
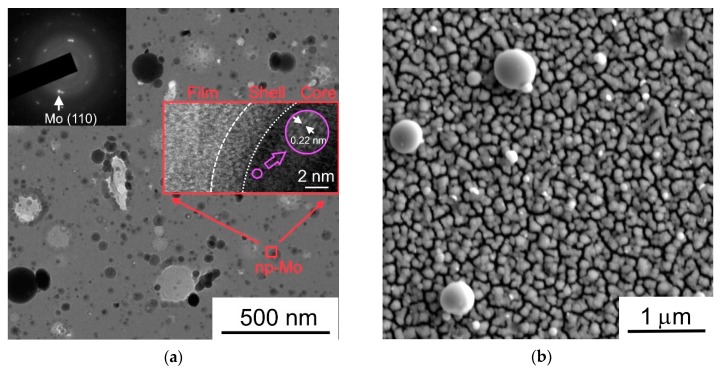
(**a**) TEM image and SAED pattern for thin MoS_3_/np-Mo film; (**b**) SEM image of MoS_3_/np-Mo// WO_3−*y*_ catalyst formed by PLD on FTO substrate. Insert in (**a**) shows the high resolution TEM of Mo nanoparticle covered with MoS_3_ shell. Submicron MoS*_x_* particles in (**b**) were formed due to splashing of MoS_2_ target under pulsed laser ablation.

Figure 9a shows the TEM/SAED patterns of the MoS_3_/np-Mo film, which was used for the preparation of hybrid MoS_3_/np-Mo// WO_3−*y*_ nanocatalyst. The MoS_3_/np-Mo film had an amorphous matrix, and the rounded particles of nanometer (~10–50 nm) and sub-micrometer (≥100 nm) sizes adhered quite well to thin MoS_3_ film. The SAED pattern measured in a local area with a particle of darkest contrast indicated the single crystalline structure of Mo nanoparticles. On high-resolution, the TEM image of a separate Mo nanoparticle, atomic planes with an interplanar distance of ~0.22 nm, which is characteristic of Mo (110), were observed. These particles were covered with a thin amorphous shell. An additional EDSX study of these films has shown that the composition of amorphous matrix and that of a shell around Mo core are similar. Taking into account the results of RBS study of thin MoS_3_ film, this study suggested that shells around Mo nanoparticles also had MoS_3_ composition. 

The deposition of thin MoS_3_/np-Mo film influenced insignificantly on the morphology of the WO_3−*y*_ underlayer. Figure 9b shows the SEM image of the MoS_3_/np-Mo// WO_3−*y*_ nanocatalyst in which the metal oxide film was deposited on the FTO substrate at air pressure of 40 Pa and this film was not annealed before the MoS_3_/np-Mo film deposition. In comparison with the SEM image in Figure 5a, only a small number of single particles of submicron size appeared on the surface of WO_3−*y*_ film after the PLD of a thin MoS_3_/np-Mo film.

### 3.3. Electrocatalytic and Photoelectrocatalytic Properites of WO_3−y_, MoS_3_/np-Mo and MoS_3_/np-Mo// WO_3−y_ Films

**Figure 10:**
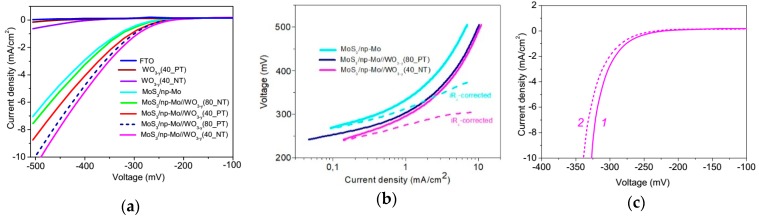
Electrocatalytic performance of FTO, WO_3−*y*_//FTO, MoS_3_/np-Mo//FTO and MoS_3_/np-Mo//WO_3−*y*_ //FTO samples: (**a**) linear sweep voltammetry (LSV) curves, (**b**) Tafel plots and (**c**) iR-corrected LSV curves that were measured before (1) and after 2000 cycles (2) of CV testing for the sample with the best electrocatalytic performance (MoS_3_/np-Mo//WO_3−*y*_ //FTO (40_NT)). The WO_3−*y*_ films was prepared by PLD at two air pressure (40 and 80 Pa) without (NT) and with posttreatment (PT) in air at 500 °C.

Figure 10a shows the results of studies of the electrocatalytic properties of the FTO, WO_3−*y*_//FTO, MoS_3_/np-Mo//FTO, and MoS_3_/np-Mo//WO_3−*y*_//FTO samples that were prepared under different conditions. The LSV curves indicated the benchmark activities of the films because both the apparent geometric area and the catalyst loading are known. These LSV measurements have revealed that a bare FTO substrate and all metal oxide films on the FTO substrate exhibited a low catalytic activity in HER. The thermal post treatment of WO_3−*y*_ films resulted in an increase of their resistance to electric current transport. The resistance R_s_ of metal oxide layers was ~30 Ω before and it became ~50 Ω after annealing. The first value coincides with the resistance R_s_ estimated for the FTO substrate. The increase of R_s_ after thermal posttreatment resulted in a decrease of HER efficiency of WO_3−*y*_.

More noticeable improvements of the HER activity was observed for a thin MoS_3_/np-Mo film deposited on the FTO substrate. Due to the MoS_3_/np-Mo film deposition, an overvoltage *U*_1_, which was required to achieve a current density of 1 mA/cm^2^, decreased (in absolute value) from −800 mV (the bare FTO) to −330 mV. The formation of metal oxide underlayer caused the additional enhancement of the electrocatalytic activity of the MoS_3_/np-Mo film. However, the performance of the hybrid MoS_3_/np-Mo//WO_3−*y*_ nanocatalysts depended on structural characteristics of the metal oxide underlayer. The best electrocatalytic performance in HER with U_1_ ≈ −291 mV was registered for the hybrid catalyst containing the WO_3−*y*_ film that was prepared by PLD at dry air pressure of 40 Pa without annealing. The thermal posttreatment of this WO_3−*y*_ underlayer caused a deterioration of the HER activity of hybrid nanocatalyst. Conversely, for the hybrid nanocatalyst containing the WO_3−*y*_ film, which was prepared by PLD at 80 Pa, the thermal posttreatment of metal oxide caused an improvement of the electrocatalytic properties and a decrease of *U*_1_ from −324 to −300 mV. 

The Tafel plots for some samples are shown in Figure 10b. To obtain the linear-type dependence of Tafel curves, the iR_s_ correction of LSV measurements should be made. For MoS_3_/np-Mo//FTO sample after the iR_s_ correction, the Tafel slope was ~62 mV/dec and it decreased to 40 mV/dec when a WO_3−*y*_ nanostructured layer was formed between the thin MoS_3_/np-Mo catalytic film and the FTO substrate. This suggests that the WO_3−*y*_ layer could influence synergistically on the HER catalytic activity of thin MoS_3_ film. The Tafel slopes indicated that for all samples, the HER reaction occurs via a Volmer–Heyrovský mechanism. However, the influence of the reactions of the proton adsorption and the electrochemical desorption of hydrogen on the HER reaction kinetics is different.

The stabilities of the prepared films were tested by continuously cycling the voltage between +100 and −350 mV with a scan rate of 50 mV/s. For all samples, nearly identical LSV curves were detected after the first and 2000th cycles of CV testing. The result of CV testing for the MoS_3_/np-Mo//WO_3−*y*_//FTO (40_NT) sample is shown in Figure 10c. This result indicates a very low loss of catalytic performance and a satisfactory stability of the electrocatalyst prepared on the FTO substrate by the PLD process.

Figure 10c shows that an onset potential of obtained MoS_3_-containing electrocatalysts was approximately −210 mV for a loading of ~4.5 μg/cm^2^. This low MoS_3_ loading was applied to reveal the synergistic interaction of MoS_3_ with WO_3−y._ The authors own experience and literature search reveal that the HER performance of the MoS*_x_*-based nanocatalysts depends on both the material of support and the catalyst loading [21,32,52]. For the MoS*_x_*-based catalytic films (including MoS_3_) deposited on FTO substrates, an onset potential of HER is obviously inferior to the films deposited on a glassy carbon substrate. The optimal loading of the MoS_3_ catalyst prepared by chemical synthesis on FTO substrates is approximately 53 μg/cm^2^ [52]. For an FTO electrode with a loading of 53 μg/cm^2^ MoS_3_, the current densities in 1.0 M H_2_SO_4_ solution were 0.18 and 1.1 mA/cm^2^ at voltages of 150 and 200 mV, respectively. The Tafel slopes were in the range of 52 to 61 mV/dec. In the case of lower loading (e.g., 13 μg/cm^2^), the HER performance of this MoS_3_ catalyst was significantly worse than that of MoS_3_ catalyst with optimal loading. For low-loading MoS_3_ catalysts, the onset potential of HER was larger than 200 mV. This indicates that the HER performance of the MoS_3_/np-Mo catalyst obtained by PLD is not inferior to that of amorphous MoS_3_ catalysts obtained by chemical synthesis.

The LSV curves shown in Figure 10a were measured under combined illumination of natural and laboratory sources of light. The LSV measurements in the dark indicated that photo-assisted processes under such illuminations weakly influenced on the HER activity of the prepared samples. 

**Figure 11:**
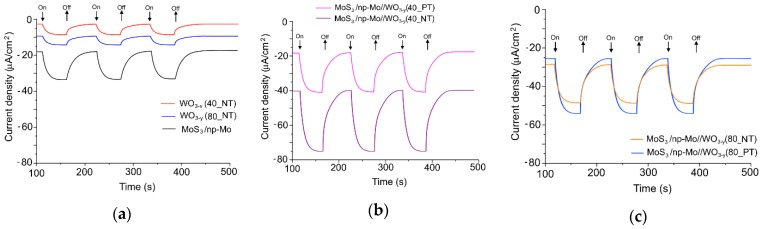
Photo-initiated hydrogen evolution reaction (HER) activity of prepared on FTO samples (measured at zero potential in 0.5M H_2_SO_4_ solution): (**a**) single WO_3−*y*_ and MoS_3_/np-Mo films; (**b**) hybrid MoS_3_/np-Mo//WO_3−*y*_ nanocatalysts containing the WO_3−*y*_ film deposited at air pressure of 40 Pa; (**c**) hybrid MoS_3_/np-Mo//WO_3−*y*_ nanocatalysts containing the WO_3−*y*_ film deposited at air pressure of 80 Pa.

Figure 11 shows the photo-assisted electric current pulses which occurred due to the on/off flux of light of Xe lamp during electrochemical HER with different prepared samples. The measurements were carried out after current stabilization under laboratory conditions of illumination. The HER activity of the WO_3−*y*_ film deposited at 80 Pa was slightly higher than that of the WO_3−*y*_ film deposited at 40 Pa. The WO_3−*y*_ catalysts on FTO substrate are obviously inferior to MoS_3_/np-Mo//FTO sample (Figure 11a). However, the highest photoelectrocatalytic activity was observed for MoS_3_/np-Mo//WO_3−*y*_//FTO sample that contained the metal oxide film prepared by PLD at air pressure of 40 Pa (Figure 11b). For this sample, the current density reached ~80 μA/cm^2^ under illumination. The thermal posttreatment of the metal oxide film caused the decrease of the HER activity of this hybrid catalysts and the current density under illumination was reduced by half.

The photoelectrocatalytic performance of the hybrid nanocatalyst that contained the metal oxide layer prepared by PLD at air pressure of 80 Pa was lower than that of the nanocatalyst with WO_3−*y*_ layer deposited at 40 Pa without annealing (Figure 11c). The thermal posttreatment insignificantly influenced the photoelectrocatalytic HER activity of these samples and this activity was slightly higher than that of the hybrid nanocatalyst containing the deposited at 40 Pa and annealed WO_3−*y*_ film. 

## 4. Discussion

**Figure 12:**
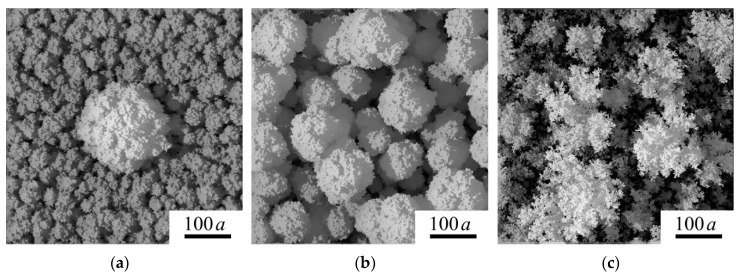
Top views of surface morphology for the films modelled by kinetic Monte Carlo (KMC) in (**a**,**b**) ballistic and (**c**) diffusion deposition regimes. The film thicknesses are (**a**) 100 *a* and (**b**,**c**) 400 *a*; *a* is the size of structural blocks used in the models. (**a**) illustrates the spherical nanoparticle with a diameter 72 *a* coated with a shell as a result of film deposition; (**b**,**c**) show the films deposited on pristine substrates.

An understanding of the film growth mechanisms during the PLD at various background gas pressures has been provided by the KMC simulations. Figure 12a,b show the model structures that can be formed if the ballistic mechanism of film growth is dominant. In order to this process of film growth proceeds, the flux of particles (atoms or clusters) should have sufficiently large kinetic energy of directed motion at the stage of approaching the substrate. This deposition mode is most likely at air pressure of approximately 40 Pa. The measured ion pulse indicated that both the high-speed component of the atomic flux and the component resulting from the scattering of the atomic flux by the molecules of the background gas were deposited. The scattering of laser ablated atoms by gas molecules results in the broadening of an angular diagram of the incident flux of atoms. The ballistic deposition causes the development of cauliflower-like morphology of the films. These films consist of closely standing columns, the formation of which is associated with a self-shadowing effect.

A comparison of Figure 12a,b shows that the transverse crown size of an individual cauliflower column depends on the deposition time of the film (thickness of the film). The relatively dense packing of columns became looser (porous) when the thickness of the model films increased from 100 *a* up to 400 *a*. For the thicker film, the lateral size of the crown can reach 100 *a*, and a good agreement of model morphology with the experimental structure is realized in the case if *a* is ~0.8 nm. For this value of *a*, the thickness of the model film can be approximately 320 nm, that quiet well coincides with the thickness of the experimental films (Figure 5). It should be noted, a characteristic size of the WO_6_ octahedra is approximately 0.8 nm, and these octahedra are the main structural elements of WO_3−*y*_ films. The octahedrons are probably formed due to the local ordering of O atoms that surround a W atom falling onto a substrate/film. In the amorphous structure of WO_3−*y*_ film, a distorted ReO_3_-type unit in which corner-sharing, distorted, and tilt WO_6_ octahedra are connected in the 3D net [9].

Figure 12a illustrates the formation of a shell on the nanoparticle caused by the deposition of atomic flux. If the nanoparticle was deposited at the initial stage of model film formation, then its size noticeably increased as a result of the sell formation. Therefore, when the core size is 72 *a* (~56 nm), the size of the nanoparticle in the plane increases to ~150 *a* due to the shell growth. The thickness of the deposited model film was ~100 *a*.

Figure 12c shows the change in the morphology of the model film in the case when the diffusion mechanism of deposition of the structural elements is realized at higher pressures of background gas. An adequate correlation of morphology of model film with that of the web-like morphology of the experimental WO_3−*y*_ film obtained at an air pressure of 80 Pa was achieved at *a* ~1 nm. This suggests that W–O clusters could be formed at the stage of the approach of the W atom to the substrate. According to the measurement of the ion signal for a laser plume, only low-speed atoms scattered in collisions fall on the substrate. The relatively slow motion of the W atom in a fairly dense reaction gas medium favors the formation of clusters [39,42]. Plasma chemistry governs the interaction of W atoms with oxygen in the plume and leads to the direct formation of W–O clusters [42]. An effective activation/dissociation of O_2_ facilitating metal–oxygen molecules formation during the reactive PLD in O_2_-containing background gas was revealed in [53].

Thus, experimental studies and modelling have shown that the PLD of WO_3−*y*_ films at air pressures of 40 and 80 Pa are favored by the nanostructuring and opening of the structure. An increase of the effective surface area of the films can be realized. The laser plume studies revealed that the PLD of metal oxide films at air pressure of 40 Pa initiates the bombardment of the substrate/film by high-speed atoms. This suggests that adhesion of these films with FTO substrates should be better than the films deposited at a higher air pressure, since the growth of the WO_3−*y*_ films at air pressure of 80 Pa proceeds without the high energy particle bombardment. A weak adhesion is usually accompanied by increased contact electrical resistance. 

Electrical conductivity is one of the crucial factors of the electrochemical activity of the prepared films. For the WO_3−*y*_ films, it depends on the concentration of oxygen vacancies which facilitate the formation of energetic levels in the energy gap for electron transport through the n-type WO_3−*y*_ [9]. MRS studies have shown that annealing caused the ordering of the local structure and even crystallization of metal oxide films. These structural modifications could have negative effects on the electrical properties of WO_3−*y*_. A decrease in the concentration of vacancies could result in a decrease in conductivity, the ordering and crystallization affects the band gap, as well as the mobility and lifetime of the carriers. Probably, due to the competition between these processes, this study revealed a multidirectional effect of annealing on the electrocatalytic properties of MoS_3_/np-Mo//WO_3−*y*_ hybrid films containing the metal oxide films deposited at 40 and 80 Pa. However, regardless of the posttreatment of the WO_3−*y*_ films, the electrocatalytic activity of MoS_3_/np-Mo//WO_3−*y*_ films was higher than that for MoS_3_/np-Mo films.

KMC modelling indicated the effective shell growth on the nanoparticles in the conditions that were realized during the PLD processes of MoS_3_/np-Mo films. As a result, for the MoS_3_/np-Mo//WO_3−*y*_ hybrid films, the thin catalytic MoS_3_ film was supported by WO_3−*y*_ and locally by nanoparticles of Mo. A possible mechanism for the formation of spherical metal nanoparticles during pulsed laser ablation of transition metal dichalcogenides targets was considered by the authors earlier in [36]. Briefly, during ablation of such targets, the predominant evaporation of chalcogen atoms occurs. Therefore, the formation of a thin metal film on the surface of the target is possible. An explosive evaporation of this metal film under laser irradiation results in the formation of nano-droplets, which assist the deposition of vapor on the substrate. The selective self-sputtering of chalcogen atoms from the deposited film should be suppressed by the deposition in a background gas. Then, the matrix of the films contain an increased concentration of chalcogen atoms (*x* > 2). Furthermore, in order to increase the concentration of chalcogen atoms, it is important to enhance the efficiency of the saturation of the film by chalcogen atoms from a S-containing vapor that accumulates in the deposition chamber during PLD.

**Figure A1:**
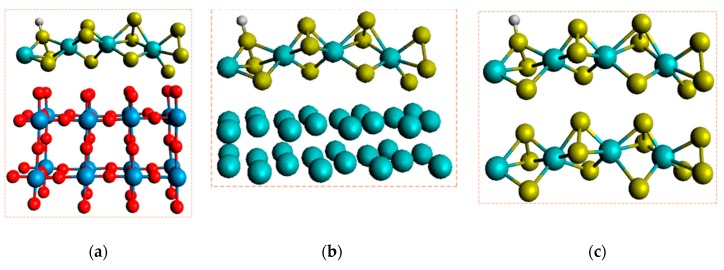
Local structures of simulated clusters: (**a**) MoS_3_/WO_3_; (**b**) MoS_3_/Mo; (**c**) MoS_3_/MoS_3_. Color scheme: Mo: light blue; S: yellow; W: dark blue; O: red; hydrogen: gray. One period of crystal structure is shown.

**Figure A2:**
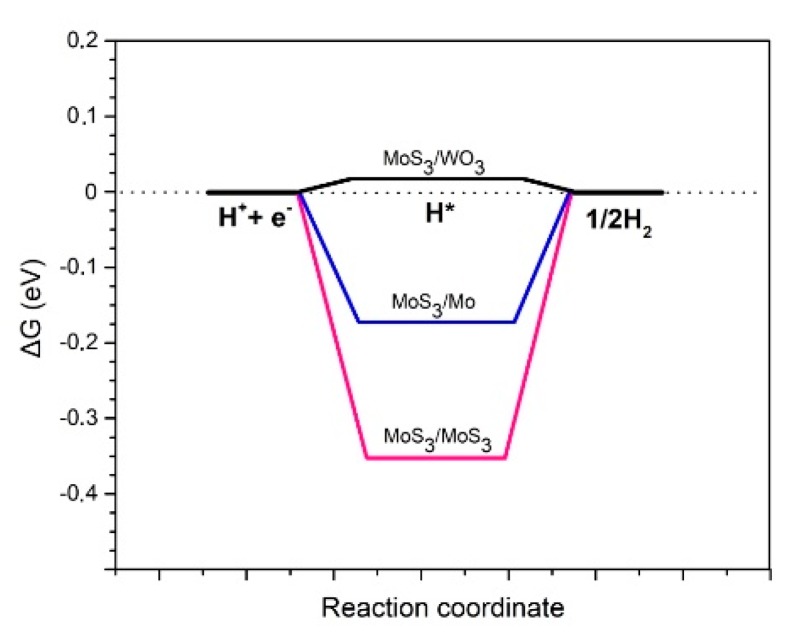
Calculated energy diagram of HER process for different nano-catalysts: MoS_3_/WO_3_, MoS_3_/Mo, and MoS_3_/MoS_3_.

Figure A1 shows the models of atomic clusters used for DFT calculation of the Gibbs free energy for hydrogen adsorption on the surface of prepared films. The MoS_3_/WO_3_ and MoS_3_/Mo hybrids as well as the binary MoS_3_/MoS_3_ were analyzed and the free energy diagram for hydrogen adsorption at the equilibrium potential for these combinations was calculated (Figure A2). The DFT calculations show that the chemical interaction of thin MoS_3_ film with WO_3_ and Mo facilitates the enhanced electrocatalytic activity of S atoms located on the surface of MoS_3_ thin film. In the case of hydrogen adsorption on S atoms on the surface of MoS_3_/WO_3_ hybrid, the calculated value of Δ*G_H_* is the lowest (~0.02 eV). For the combination of MoS_3_/Mo, the Δ*G_H_* is ~−0.17 eV. This value is larger than the MoS_3_/WO_3_ hybrid, but it remains quite small compared to the Δ*G_H_* for the combination MoS_3_/MoS_3_ (~−0.35 eV). The latter combination most adequately models the chemical state of the surface of the MoS_3_ films on the FTO substrate. The local thickness of the MoS_3_ films on a smooth surface of FTO substrate is much larger than the 3D porous WO_3−*y*_. There is a proven correlation between the HER activity of catalysts and their Δ*G_H_* values [54]. With a small Δ*G_H_*, the processes of formation and desorption of H_2_ molecule proceed most rapidly. Therefore, in accordance with the DFT calculation, the HER efficiency of a thin MoS_3_ film on the WO_3−*y*_ is higher than that of a thicker MoS_3_ film on FTO. Moreover, Mo nanoparticles do not negatively affect the HER efficiency of MoS_3_.

The nanostructured films containing WO_3−*y*_ and MoS_2_ phases are currently used to create photoanodes for effective photo-electrochemical water oxidation reaction (oxygen evolution reaction, OER) [2,3,9,55,56,57]. This is due to the adequate optical properties of these semiconductor materials and their energy band structures. The valence band (VB) level of these materials locates below the O_2_/H_2_O level indicating the thermodynamic ability for OER due to the participation of photo-generated holes in the OER process. As noted above, the use of WO_3−*y*_ for photocatalytic hydrogen evolution has a principle limitation due to the peculiarity of the energy band structure. Therefore, for the effective use of the beneficial properties of the MoS_2_ and WO_3−*y*_ semiconductor materials for photo-HER, more complex heterojunction configurations, for example, by adding a third component (CdS), were created [58].

The authors desired to test a simpler configuration by combining with H_2_-evolution photocatalyst MoS_3_ to construct a two-step excitation system (like the Z-scheme) [9,59,60]. The energy band structure of amorphous MoS_3_ is not well studied. Moreover, the energy levels of conduction band (CB) and VB for MoS_3_, as well as for WO_3−*y*_, depend on the chemical composition and local structure of these semiconductors. It is reasonable to propose that the character of the energy band structure of MoS_3_ is in many respects like that of MoS_2_, i.e., the CB level is located above the H^+^/H_2_ potential and the energy band gap (~1.5 eV) is slightly less than that of MoS_2_ sheets (1.8 eV). The advantage of MoS_3_ over MoS_2_ is their higher electronic conductivity, additional sulfur species on the surface, which can provide enhanced electrochemical properties [61,62]. The band gap of WO_3−*y*_ is ~2.8 eV, and the CB level of WO_3−*y*_ locates below the H^+^/H_2_ level. The band gap of FTO is ~3.8 eV and the CB level of FTO locates near the CB level of WO_3−*y*_ [9,63].

In dark conditions, the electrons of the CB of MoS_3_ tend to move to the CB of WO_3−*y*_ due to the difference of their energy in these semiconductors. As a result, an internal electric field at the MoS_3_/WO_3−*y*_ interface is formed, and it is directed from MoS_3_ to WO_3−*y*_. The Z-scheme photocatalytic mechanism of photo-assisted HER for MoS_3_/WO_3−*y*_ hybrid proceeds by the initial light absorption in both semiconductors. The photogenerated electrons in the CB of WO_3−*y*_ combine with photogenerated holes in the VB of the MoS_3_ (electron-hole recombination), and the photogenerated electrons in the CB of MoS_3_ are left in this band to promote the HER reaction. The energy barrier prevents the transfer of photogenerated electrons from MoS_3_ to WO_3−*y*_. The holes formed in WO_3−*y*_ film under light illumination recombine with electrons at the WO_3−*y*_ /FTO interface. The internal electric field prevents the transport of photogenerated holes from WO_3−*y*_ to MoS_3_. The compared studies of MoS_3_/np-Mo//WO_3−*y*_ nanostructured films containing amorphous, slightly ordered and crystalline forms of WO_3−*y*_ revealed that the efficiency of photoelectrochemical HER for this hybrid largely depends on the local structure of the metal oxide film. The search for the optimal local structure of the metal oxide layer that provides the highest performance of the MoS_3_/np-Mo//WO_3−*y*_ hybrid, as well as the determination of maximum photo-assisted HER activity achievable by PLD of this combination, are currently under study.

## 5. Conclusions

This study proposed and validated a simple and efficient laser-based technique to form (photo)electrocatalytic nanohybrids for effective HER by simply depositing 3D porous WO_3−*y*_ and MoS_3_/np-Mo catalytic film. The required nanostructure and chemical state of the formed layers were realized by selecting the modes of pulsed laser ablation of the WO_3_ and MoS_2_ targets and by optimizing the conditions for the transport and deposition of the laser plume in the background gas. The KMC simulation provided an understanding of the growth mechanisms at various background gas pressures. According to the experimental results and DFT calculation, these MoS_3_/np-Mo//WO_3−*y*_ hybrids exhibit enhanced (photo)electrocatalytic properties superior to that of any pristine one (MoS_3_/np-Mo or WO_3−*y*_) with an equal loading on the FTO substrate. The improved electrochemical HER activity for the hybrid catalysts can be attributed to the synergistic effects from the dense catalytic sites at the MoS_3_ surface, good dispersion of the MoS_3_ and Mo nanoparticles on 3D porous structure of WO_3−*y*_, good electrical contact and chemical bonding between MoS_3_, Mo nanoparticles and WO_3−*y*_ layers, as well as between the porous WO_3−*y*_ and the FTO substrate. The photoelectrochemical HER activity of MoS_3_/np-Mo//WO_3−*y*_ hybrids proceeds due to the generation of electron-hole pairs in the MoS_3_ semiconductor under visible light illumination and subsequent holes separation/transfer into the photo-exited WO_3−*y*_ underlayer in accordance with a direct Z-scheme photocatalytic mechanism.

## Appendix A

To demonstrate the role of the substrate on the catalytic activity of thin MoS_3_ films, DFT simulation were performed of several simplified atomic structures using the Quantum ESPRESSO package [48]. The details of the simulation are as follows.

The Perdew-Burke-Ernzerhof (PBE) of the projector augmented-wave (PAW) method was used as the exchange-correlation function.

The geometry optimization was carried out using the Broyden-Fletcher-Goldfarb-Shanno (BFGS) algorithm. The kinetic energy cutoff was 60 Ry, while the charge density cutoff was 500 Ry. The first irreducible Brillouin zone was sampled by 4 × 4 × 1 set of *k*-points generated through the general Monkhorst-Pack scheme. Two bottom atomic layers were fixed, while all other layers and adsorbate could relax until the total energies converged up to 1.0 × 10^−5^ and the force convergence criterions were set as 0.07 eV/Å.

The Gibbs free energy for hydrogen adsorption was calculated using the relation ∆*G_H_* = ∆*E_H_* + ∆*E_ZPE_* − *T*Δ*S_H_*. The following equation was used to calculate the hydrogen adsorption energy (∆*E_H_*):

$$\Delta E_{H}=\frac{E(catalyst+nH)-E(catalyst)}{n}-\frac{E(H_{2})}{2}$$

Here, *E(catalyst+nH)* is the total catalyst energy with *n* adsorbed hydrogen atoms, *E(catalyst)* is the total catalyst energy, *E(H_2_)* is the total hydrogen molecule energy in a gas phase. ∆*E_ZPE_* is the zero-point energy difference between the adsorbed state of the system and the gas phase state. Δ*S_H_* is the entropy difference between the adsorbed state of the system and the gas phase standard state. The entropy of hydrogen adsorption can be calculated as Δ*S_H_* ≈ 1/2 ($S_{}{}_{H_{2}}^{\circ }$), where $S_{}{}_{H_{2}}^{\circ }$ is the entropy of gas phase H_2_ at standard conditions [64].

Figure A1a shows the model structure consisting of MoS_3_ clusters deposited on the WO_3_ film. This combination was used for periodic plane wave DFT calculations to reveal the synergistic influence of the WO_3_ underlayer on the catalytic activity of thin MoS_3_ film. It was suggested that the local structure of the catalytic MoS_3_ film is formed by linear chains of atoms and it consisted of MoS_6_ blocks in which three S atoms form bonds with two neighboring Mo atoms [65]. The MoS_3_ cluster was placed on the (200) plane of monocline phase of WO_3_. In the process of geometry optimization, the atomic position of the lower WO_3_ layer was fixed. It was proposed that local structures of WO_3_ and WO_3−*y*_ clusters do not differ significantly.

The influence of Mo nanoparticles on the HER activity of MoS_3_ due to the chemical interaction was studied by DFT simulation of MoS_3_/Mo cluster combination (Figure A1b). For this, the MoS_3_ chain was used which consisted of 4 segments and MoS_3_ clusters were located above two (100) atomic planes of Mo.

The chemical state of the thicker MoS_3_ film deposited on the FTO substrate was simulated by constructing the MoS_3_/MoS_3_ combination which excluded the effective interaction of the surface layer of the catalyst with the FTO substrate. This study used a cell which had 4 MoS_3_ segments lying along the [100] direction above the base (Figure A1c). A distance of 8 Å between adjacent chains allowed the significant reduction of their interaction and the procurement more correct numerical results.

The simulation cell size was taken corresponding to the size of model structures.

The calculated free-energy changes during hydrogen evolution proceeded by the Volmer–Heyrovsky reaction are shown in the energy diagram in Figure A2.
